# Applications of Continuous Glucose Monitoring in Cognitive Research in Diabetes: a Scoping Review

**DOI:** 10.1007/s11892-026-01638-1

**Published:** 2026-08-06

**Authors:** Jeeyeon Kim, Jayeong Kim, Heather Cuevas, Anna Kratz

**Affiliations:** 1 Department of Physical Medicine and Rehabilitation, University of Michigan, 2800 Plymouth Road, North Campus Research Complex Building 16, Ann Arbor, MI 48109 USA; 2 Seoul National University College of Nursing, Seoul, Korea; 3 School of Nursing, University of Texas at Austin, 1710 Red River St, Austin, TX 78712 USA

**Keywords:** Cognitive function, Continuous glucose monitoring, Diabetes, Scoping review

## Abstract

**Purpose of Review:**

Continuous glucose monitoring (CGM) has increasingly been incorporated into studies examining cognitive function in people with diabetes. This scoping review systematically maps how CGM has been applied in cognitive research among individuals with diabetes to characterize current methodological approaches, identify key knowledge gaps, and inform future research.

**Recent Findings:**

A scoping review was conducted following Arksey and O’Malley’s framework. A systematic search of five electronic databases (PubMed, CINAHL, Cochrane Library, EMBASE, PsycINFO) was performed. Cognitive outcomes were extracted and mapped to the International Classification of Functioning, Disability and Health framework. Twenty-one studies met the inclusion criteria. CGM data completeness was consistently high, with 84–100% of expected CGM data captured, supporting the feasibility of intensive glucose monitoring in cognitive research in diabetes. Considerable heterogeneity was observed in cognitive domains and assessment tools. Most studies evaluated executive function, memory, and attention, whereas psychomotor, language, and perceived cognitive functions were less frequently assessed. More than 10 distinct instruments were used to assess memory and executive function. Across studies, similar cognitive domains were examined regardless of the specific CGM metrics employed.

**Summary:**

Research examining CGM and cognitive function in diabetes is characterized by substantial methodological variability in both CGM metrics and cognitive assessments. These findings underscore the need for greater standardization to improve cross-study comparability and to advance CGM-informed cognitive research, including the development of personalized interventions to support cognitive health through optimized glucose management in people with diabetes.

**Supplementary Information:**

The online version contains supplementary material available at 10.1007/s11892-026-01638-1.

## Introduction

Diabetes mellitus is a chronic metabolic condition characterized by impaired insulin production or utilization, leading to persistent dysregulation of blood glucose levels [1]. Beyond its well-established vascular and metabolic complications, diabetes substantially increases the risk of cognitive impairment, with prevalence estimates reaching up to 58% of affected individuals [2, 3]. Cognitive deficits in diabetes span multiple domains, including memory, attention, executive function, language, and visuospatial abilities, [4] and carries significant clinical consequences. These deficits interfere with social functioning, [5] undermine diabetes self-management behaviors [6], and ultimately elevate the risk of complications and reduce quality of life.

The mechanisms linking diabetes to cognitive decline are multifactorial, involving insulin resistance, chronic inflammation, oxidative stress, and vascular injury [4]. Among these, glycemic control has emerged as a particularly critical determinant of cognitive health [7]. Uncontrolled glucose and hyperinsulinemia disrupt neuronal metabolism, intensify oxidative stress and inflammation, and impair synaptic and mitochondrial function.[8] Over time, these processes compromise neuronal integrity, leading to structural brain damage and cognitive deterioration [8]. Traditionally, glycated hemoglobin (HbA1c) has served as the primary biomarker of long-term glycemic control. However, HbA1c provides only an average estimate over 3 months and fails to capture short-term fluctuations or daily variability, which may exert independent and potentially stronger effects on cognitive outcomes.

Continuous glucose monitoring (CGM) addresses these limitations by providing real-time, high-resolution glucose profiles and enabling the assessment of dynamic metrics such as time in range (TIR; percentage of time within a target glucose range), mean amplitude of glucose excursion (MAGE; magnitude of major glucose swings), and coefficient of variation (CV; relative glucose variability). CGM is increasingly regarded as the gold standard for characterizing glycemic patterns in both clinical care and research [9, 10] and offers a unique opportunity to elucidate the complex relationship between glucose dynamics and cognitive outcomes in diabetes. Although this field remains nascent, emerging evidence suggests that glucose variability—captured by metrics such as CV and postprandial glucose excursions—may contribute to cognitive decline independently of average glucose levels.[11, 12].

In recent years, the number of studies applying CGM-derived metrics to examine glucose–cognition relationships in diabetic populations has grown rapidly. While this expanding body of work underscores the potential importance of glucose dynamics for cognitive health, the existing evidence remains fragmented due to substantial methodological heterogeneity and the lack of standardized guidelines for CGM metrics and cognitive assessment.[12] Moreover, the feasibility of integrating CGM into cognitive research and adherence to CGM protocols have not been comprehensively evaluated. Accordingly, a scoping review is warranted to systematically map how CGM has been applied in cognitive research among individuals with diabetes.

The objectives of this scoping review are to (1) evaluate the feasibility of CGM implementation in cognitive research and (2) characterize the study designs, population features, CGM-derived metrics, cognitive domains, and cognitive assessment tools used across the existing literature. By synthesizing this emerging evidence, this review seeks to clarify current methodological trends, identify key knowledge gaps, and inform priorities for future research in this evolving field of diabetes and cognitive health.[13].

## Methods

Given the heterogeneity in measurements and reported outcomes of existing studies, a scoping review was selected as the appropriate approach to provide a comprehensive overview of the current evidence landscape. We followed the five-stage framework developed by Arksey and O’Malley and reported findings in accordance with the PRISMA extension for Scoping Reviews.[14].

Stage 1: Identifying the research question.

This review was guided by the following research questions:

1. What study designs and methodological approaches have been employed in studies that incorporated CGM to examine cognitive function in diabetes?
2. What population characteristics (e.g., type of diabetes, age group, race/ethnicity, clinical setting) have been examined?
3. Which CGM metrics and cognitive domains or tools have been applied and how they have been paired?
4. How feasible CGM integration has been, as reflected by adherence and data completeness?

Stage 2: Identifying relevant studies.

We searched PubMed, CINAHL, Cochrane Library, EMBASE, and PsycINFO for studies published through August 2025 using database-specific combinations of terms related to continuous glucose monitoring, cognition, and diabetes, developed in consultation with a librarian. Full search strategies are provided in Supplementary Table S1. Additional studies were identified via Google Scholar and reference list screening.

Stage 3: Study selection.

Records were imported into EndNote 21, and duplicates removed. Two reviewers (JK and JK) independently screened titles/abstracts and full texts, with disagreements resolved by a third reviewer (HC). Eligible studies included adults with diabetes that used CGM and assessed cognitive function. No restrictions were placed on study design, diabetes type, or setting. Studies involving pediatric samples, neuroimaging-only outcomes, or non–peer-reviewed or non-original publications were excluded.

Stage 4: Charting the data.

Two reviewers (JK and JK) independently extracted data using a standardized table (Table 1), including study characteristics, participant demographics, CGM implementation details (device, duration, metrics), and cognitive outcomes. Definitions of CGM metrics are provided in Supplementary Table S2. Discrepancies were resolved by consensus. Consistent with scoping review methodology, no formal assessment of methodological quality or risk of bias was undertaken, as the purpose was to provide a descriptive overview of the available literature rather than a critical appraisal of study rigor [36].

**Table 1:** Summary of the included studies

	First Author, Year, Country	Study Type	Study Design	Setting	Study Aims	Population			CGM				Cognitive Outcome
						Diabetes Type (sample size)	Age (mean)	Race/Ethnicity	Sensor Brand	Monitoring Duration	Metrics	Data Availability(%)	Measurement Tool (Cognitive domain)
1	Boureau, 2023, France [15]	Observational	Prospective	Community	To determine the frequency and predictors of hypoglycemia in older patients with insulin-treated T2D	Insulin-treated T2DM patients aged 75 years and older (*n* = 155)	81.5	NR	Abbott FreeStyle Libre Pro	28 days	Mean glucose CV TAR TBR TIR	94.8	MMSE (Global cognitive function) FAB (executive function)
2	Chaytor, 2018, United States [16]	Observational	Cross-sectional	Community	1. To characterize the degree of cognitive dysfunction of older adults with longstanding type 1 diabetes 2. To identify biomedical factors associated with clinically significant cognitive impairment	T1DM aged ≥ 60 with cognitive impairment (*n* = 96) and normal cognition (*n* = 105)	68.3	Non-Hispanic White (92%)	Dexcom SEVENPLUS	2 weeks	Mean glucose TAR TBR	NR	SDMT oral&written (Processing speed) TMT A&B (Executive function) HVLTR (Verbal recognition memory) GPT (Visual-motor coordination)
3	Cuevas, 2022, United States [17]	Observational	Cross-sectional	Community	To explore relationships among perceived cognitive function, glucose variability, and self-management in older adults with type 2 diabetes	T2DM aged ≥ 65 without cognitive disorder (*n* = 30)	68.5	Non-Hispanic White (33.3%) Hispanic (43.3%) African American (23.3%)	Personal CGM	2 weeks	Mean glucose SD TAR TBR % out of TIR	NR	MMQ (Perceived memory function) Barkley Deficits in Executive Functioning Scale (Perceived executive function)
4	Cuevas, 2024, United States [18]	Observational	Cross-sectional	Community	To examine the association between glucose variability, diabetes self-management, and cognitive function in type 2 diabetes	T2DM aged ≥ 50 who self-reported subjective cognitive concerns without dementia (*n* = 95)	65.6	Non-Hispanic White (44.7%) Hispanic (34.2%) Black (12.2%) Asian (3.5%)	Abbott’s FreeStyle Libre Pro	Up to 2 weeks	Mean glucose CV SD MODD MAGE TAR TBR TIR	NR	PROMIS cognition (Perceived cognitive function) TMT A&B (Attention) SCWT (Executive function) DSST (Processing speed) Immediate and Delayed Recognition (Memory)
5	Cui, 2014, Israel [19]	Observational	Cross-sectional	Community	To investigate the relationship between glycemic variability, brain volumes, and cognition in T2DM	T2DM (*n* = 43) Non-diabetic (*n* = 26) without dementia	65.4	White (76.8%) Hispanic (2.9%) African American (18.8%)	Medtronic iPro Professional CGM	3 days	Mean glucose SD MAGE TBR Multi-Scale GV	93.2	VLTR (Verbal learning and memory) ROCFT (Visual-spatial ability and visual memory function) TMT A&B (Executive function) Verbal Fluency (Executive function)
6	Dong, 2023, China [20]	Observational	Cross-sectional	Community	To investigate the relationship between key CGM-derived metrics and specific cognitive domains	T2DM aged between 40 and 80 years (*n* = 96)	61.4	NR	Abbott’s FreeStyle Libre	3 days	TIR TBR TAR CV MAGE GRI Mean glucose	NR	MMSE (Global cognitive function) MoCA (Global cognitive function) DST forward and backward (Memory) TMT A&B (Attention, executive function) BNT (Language) CDT (Visuospatial ability) VFT (Language, executive function) AVLT (Immediate, delayed, cued recall, long-delayed recognition)
7	Hawks, 2024, United States [21]	Observational	Prospective (repeated-measures EMA	Community	1. Characterizing dynamic, within-person associations between glucose and cognition 2. Examining individual differences in cognitive vulnerability to glucose fluctuations	Adults with T1DM (*n* = 200)	45.7	White (86%) Hispanic (6.5%) African American (5.5%) Asian (1%)	Dexcom G6	Up to 20 days	Mean glucose SD CV TIR TBR TAR	99	DSM (Processing speed) GCPT (Sustained attention)
8	Hoogendoorn, 2025, United States [22]	Observational	Prospective (repeated-measures EMA)	Community	1.Examining bidirectional associations between glucose metrics and cognitive function 2. exploring participant characteristics that may moderate relationships between glucose and cognitive performance	Adults with T1DM (*n* = 182)	40	Non-Hispanic White (29.1%) Hispanic (40.7%) Black (14.8%) Asian (3.8%)	Abbott FreeStyle Libre Pro	2 weeks	Mean glucose CV TIR TBR TAR TVH	93.3	Symbol search (Perceptual speed) GCPT (Sustained attention)
9	Inoue, 2025, Japan [23]	Observational	Prospective longitudinal (2-year follow-up)	Community	To investigate the association between CGM-derived glycemic control indicators and the decline in cognitive function in patients with T2DM	T2DM aged ≥ 60 without dementia & MCI (*n* = 197)	68.8	NR	Abbott’s FreeStyle Libre Pro	2 weeks	Mean glucose CV TIR TAR TBR TITR GRI	NR	MMSE (Global cognitive function) MoCA-J (Global cognitive function) DSST (Processing speed, visual short-term memory, attention, executive function and decision-making)
10	Kim, 2025, United States [24]	Observational	Cross-sectional	Community	To examine associations between physical activity, glucose variability, and cognitive function	T2DM aged 50 years and older who self-reported subjective cognitive concerns without dementia (*n* = 87)	59.2	White (60.9%) Black (20.7%) Asian (4.6%)	Abbott FreeStyle Libre Pro	Up to 2 weeks	CV	NR	TMT A&B+ SCWT +SDMT + Immediate and Delayed Recognition Test (Global cognitive function)
11	Meng, 2023, China [25]	Observational	Retrospective	clinical	To explore the correlation between glucose variability, lacune burden and cognitive function	T2DM with lacunes with cognitive function disorder(*n* = 37) and without cognitive function disorder (*n* = 107)	NR	NR	Abbott	3 days	SD CV TIR LAGE	NR	MoCA (Global cognitive function)
12	Pearce, 2012, Australia [26]	Experimental	Prospective (pre/post intervention	Community	To explore the relationship between glucose levels (pre- and post-meal spikes) via CGM and cognitive performance, before and after a weight loss diet	T2DM aged between 34 and 75 years (*n* = 44)	59.6	White (100%)	Medtronic MiniMed	40–48 h	G_max_ AUC_24_ Time spent above 12 mmol/L (T > 12)	91.4	DST forward (Short-term memory) DST backward (Working memory) IT(Speed of processing) DSST(Psychomotor speed and concentration) TMT A&B(Executive function)
13	Pyatak, 2023, United States [27]	Observational	Prospective (repeated-measures EMA)	Community	Explore whether overnight glucose variability predict next-day functioning outcomes (cognitive, physical activity, self-reported participation)	Adults with T1DM (*n* = 166)	41.0	Hispanic (39.8%) Non-Hispanic White (30.7%) Black (15.1%) Asian (4.2%)	Abbott’s FreeStyle Libre Pro	2 weeks	CV TBR TAR TIR	84.7	GCPT(Sustained attention) Symbol search (Perceptual speed)
14	Rizzo, 2010, Italy [28]	Observational	Cross-sectional	Community	To evaluate whether MAGE is associated with cognitive performance independently of main markers of sustained hyperglycemia	Older adults with T2DM without dementia (*n* = 121)	78	NR	GlucoDay	3 days	MAGE PPG	NR	MMSE (global cognitive function) TMT A&B (visuomotor speed, cognitive flexibility) DST backward (immediate recall) DST forward (immediate recall) VFT (executive function)
15	Søholm, 2024, five European countries [29]	Observational	Prospective	Community	To determine the impact of hypoglycaemia among adults with type 1 diabetes and insulin-treated type 2 diabetes on daily functioning	Adults with diabetes (T1DM, *n* = 274; T2DM, *n* = 320)	54.1	White (88.9%) Black (3.2%) Asian (2.4%)	Abbott’s Freestyle Libre 2	70 days	TIR TAR TBR	NR	PDQ (perceived cognitive function)
16	Sugimoto, 2022, Japan [30]	Observational	Cross-sectional	Community	To examine the association between CGM derived metrics and cognitive performance in older adults with T2DM	T2DM aged ≥ 70 without dementia (*n* = 100)	77.0	NR	Abbott’s FreeStyle Libre Pro	Up to 2 weeks	Mean glucose SD CV TIR TAR TBR	NR	MoCA-J (Global cognitive function) Delayed word-recall test (Memory) DSST (Processing speed) TMT A&B (Attention, executive function) LWFT (Verbal fluency) DST (Working memory & attention)
17	Sugimoto, 2023, Japan [31]	Observational	Prospective longitudinal (1-year follow-up)	Community	To examine whether CGM-derived metrics are associated with a 1-year cognitive decline in older adults with T2DM.	T2DM aged ≥ 70 without dementia (*n* = 70)	77.0	NR	Abbott’s FreeStyle Libre Pro	Up to 2 weeks	Mean glucose SD CV TIR TAR TBR	NR	MoCA-J (global cognitive function)
18	Svensson, 2025, Denmark and Netherlands [32]	Observational	Cross-sectional	Community	To investigate associations between recent real-life CGM-recorded hypoglycemia and cognitive function measured during a subsequent hyperinsulinemic-hypoglycemic clamp	Adults with T1DM (*n* = 42)	51.0	NR	Abbott’s Freestyle Libre 1	7 days	TBR Mean glucose SD CV	89.4	PASAT (Processing speed and working memory) Test of Attentional Performance (TAP)- Alertness (Processing speed), Verbal Flexibility (Attention and executive function) Working Memory (Working memory)
19	Yu, 2023, China [33]	Observational	Cross-sectional	Community	To characterize the comparative contributions of different glycemic indicators to cognitive dysfunction	T2DM aged ≥ 45 with intact cognition (*n* = 307) and MCI without dementia (*n* = 142)	58.3	NR	Medtronic	7 days	TIR SD CV MAGE TAR TBR CGI	NR	RBANS (Immediate memory, visuospatial construction ability, language, attention, and delayed memory) TMT A&B (Processing speed, executive function) SCWT (Executive function)
20	Zhong, 2012, China [34]	Observational	Cross-sectional	Community	To investigate the relationship between glucose excursion and cognitive function	T2DM patients aged ≥ 65 with no dementia (*n* = 248)	80.2	NR	Medtronic MiniMed	3 days	Mean glucose SD LAGE MAGE MODD	100	MMSE (global cognitive function) CDR (global cognitive function) GDS (global cognitive function) CDT (executive function)
21	Zuniga-Kennedy, 2024, United States [35]	Observational	Prospective (repeated-measures EMA)	Community	To examine the within-person impact of nocturnal glycemia on next day cognitive performance in adults with T1DM.	Adults with T1DM no dementia (*n* = 20)	39.8	White (100%)	Dexcom G6	15 days	TBR TAR	90	GCPT (sustained attention and response inhabitation) MOT (visuospatial working memory) DSM (psychomotor processing speed)

Symbol Digit Modalities Test (SDMT); Trail Making Test (TMT); Hopkins Verbal Learning Test-Revised (HVLTR); Grooved Pegboard Test (GPT); Multifactorial Memory Questionnaire (MMQ); The Stroop Color and Word Test (SCWT); Digit Symbol Substitution Test (DSST); Time in Range (TIR; 70–180 mg/dL); Coefficient of Variation (CV); Mean Amplitude of Glucose Excursion (MAGE); Mean of Daily Differences (MODD); Standard Deviation (SD); Mini-Mental State Examination (MMSE); Rey-Osterreith Complex Figure Test (ROCFT); Time above range (TAR; > 180 mg/dl); Time below range (TBR; < 70 mg/dl); Letter Word Fluency Test (LWFT); Complexity of Glucose Time Series Index (CGI); Glycemia Risk Index (GRI); Time In Tight Range (TITR; 70–140 mg/dL); Digit Span Test (DST); Boston Naming Test (BNT); Clock Drawing Test (CDT); Verbal Fluency Test (VFT); Auditory Verbal Learning Test (AVLT); Gradual Onset Continuous Performance Test (GCPT); Multiple Object Tracking (MOT); Time in Very High (TVH; >250 mg/dL); The largest amplitude of glucose (LAGE); Maximum postprandial peak glucose (Gmax); 24-hour area under the glucose curve (AUC_24_); Postprandial Glycemia (PPG); clinical dementia rating (CDR), global deterioration scale (GDS); Digit Symbol Matching (DSM); Frontal Assessment Battery (FAB); Repeatable Battery for the Assessment of Neuropsychological Status (RBANS); Inspection Time (IT)

Stage 5: Collating, summarizing, and reporting results.

Findings were synthesized narratively to describe methodological patterns, variability in CGM metrics, and evidence gaps. For cognitive outcomes, domains were first extracted as reported by each study. When domains were not explicitly stated in the study, we classified the primary function that each neuropsychological test was intended to assess. These domains were then mapped to the International Classification of Functioning, Disability and Health (ICF) framework, which provides a standardized structure for categorizing health-related functions.[37] Within the mental functions category, domains were further grouped into global and specific mental functions (e.g., attention, memory, psychomotor function). Semantic terms reported in the studies were consistently aligned to ICF categories; for example, “sustained attention” and “attention” were classified under attention functions, while “immediate memory” and “delayed memory” were classified under memory functions (Supplementary Table S3). Two reviewers (JK and JK) independently conducted domain mapping, with disagreements resolved by a third reviewer (AK). We calculated the proportion of studies assessing each domain and summarized assessment tools used. Perceived cognitive function measures were analyzed separately because they evaluate individuals’ subjective perceptions rather than performance-based cognitive abilities and therefore do not correspond to ICF-defined mental functions. Finally, CGM metrics were cross-tabulated with cognitive domains to identify commonly examined pairings. Figures were generated in R (version 4.4.3).

## Results

### Study and Participant Characteristics

A total of 1,589 records were identified, of which 1,342 remained after duplicate removal. Thirty-five full-text articles were reviewed, and 21 met the inclusion criteria (See supplementary material Fig. 1). Publications spanned 2010–2025, with nearly two-thirds published since 2022, reflecting rapidly expanding interest in glucose–cognition research. Studies were conducted in the United States (*n* = 8),[16–18, 21, 22, 24, 27, 35] Europe (*n* = 4),[15, 28, 29, 32] Asia (*n* = 8),[19, 20, 23, 25, 30, 31, 33, 34] and Australia (*n* = 1).[26] All samples were community-dwelling adults, except for one clinic-based cohort.[25] Most studies (*n* = 20) used observational designs, including 11 cross-sectional, [16–20, 24, 28, 30, 32–34] 8 prospective cohorts (four with ecological momentary assessment [EMA]), [15, 21–23, 27, 29, 31, 35] and one[25] retrospective analysis. Only one study used a pre–post design to evaluate the cognitive effects of a dietary weight-loss intervention.[26] Fourteen studies focused exclusively on type 2 diabetes (T2DM), [15, 17–20, 23–26, 28, 30, 31, 33, 34] six on type 1 diabetes (T1DM), [16, 21, 22, 27, 32, 35] and one included both insulin-treated T1DM and T2DM.[29] Several T2DM studies targeted older adults (≥ 60 years), [15, 18, 23, 30, 31, 34] and many excluded participants with dementia (*n* = 10).[18, 19, 23, 24, 28, 30, 31, 33–35] Sample sizes ranged from 20 to 594. Where reported, U.S. samples were predominantly White (60–100%), with limited racial and ethnic diversity.

### CGM Characteristics

CGM devices and monitoring protocols varied substantially across studies. The Abbott FreeStyle Libre was the most frequently used system (*n* = 11),[15, 17, 20, 22–24, 27, 29–32] followed by Dexcom[16, 21, 35] and Medtronic[19, 26, 33, 34] devices. One study relied on participants’ personal CGM units.[17] Monitoring durations ranged from 2 to 70 days, with most studies monitoring for 1–2 weeks (*n* = 11).[16–18, 22–24, 27, 30–33] Nine studies reported CGM wear-time completeness (84–100%), supporting feasibility.[15, 19, 21, 22, 26, 27, 32, 34, 35] Across studies, 19 different CGM-derived metrics were identified (See supplementary material Fig. 2). Range-based metrics were the most frequently reported, including time below range (TBR; *n* = 16),[15–23, 27, 29–33, 35] time above range (TAR; *n* = 14),[15–18, 20–23, 27, 29–31, 33, 35] and TIR (*n* = 13),[15, 17, 18, 20–23, 25, 27, 29–31, 33]. Seventeen studies reported at least one glycemic variability index, most often the CV (*n* = 13)[15, 17, 20–25, 27, 30–33] and standard deviation (SD; *n* = 10).[17–19, 21, 25, 30–34] Six studies[17, 19, 20, 28, 33, 34] used the MAGE to characterize the magnitude of glucose fluctuations, and two reported the Largest Amplitude of Glucose Excursions (LAGE).[25, 34] Less commonly, composite metrics such as the Glycemic Risk Index (GRI; *n* = 2)[20, 23] appeared. Postprandial-specific measures, including maximum postprandial glucose (Gmax)[26] and postprandial glycemia (PPG), [28] each appeared in one study.

### Cognitive Assessment Tools and Domains

Most studies assessed cognitive function at a single time point concurrent with CGM monitoring. Six studies included longitudinal or repeated cognitive assessments, [21–23, 27, 31, 35] four of which employed EMA to capture within-person fluctuations multiple times per day.[21, 22, 27, 35] A wide range of cognitive domains and instruments was used across studies (See supplementary material Fig. 3). Frequently assessed domains included executive function (61.9%), memory (52.4%), attention (47.6%), thought functions (i.e., processing speed; 38.1%), and intellectual functions (global cognition; 38.1%). Perceptual functions (23.8%), psychomotor functions (19%), language (14.3%), and perceived cognitive function (14.3%) were rarely examined.

**Fig. 1:**
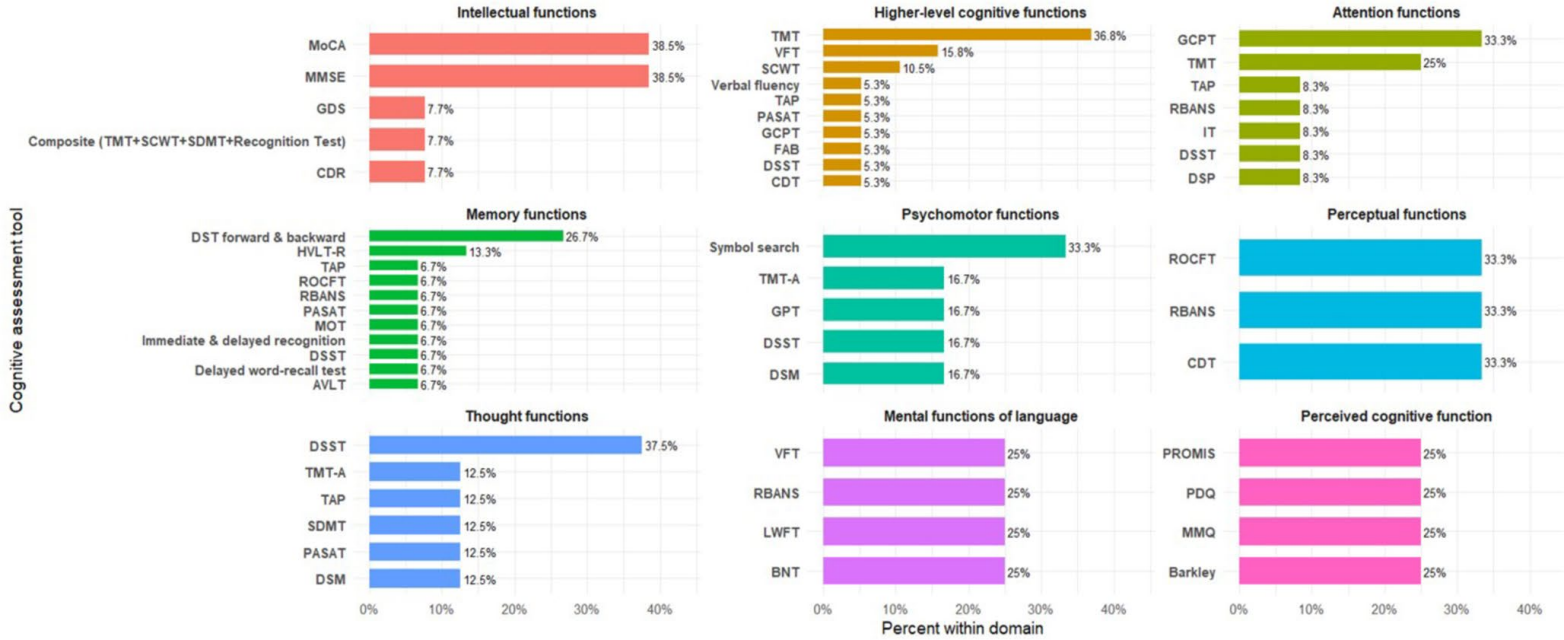
Cognitive assessment tools used within each cognitive domain

Substantial heterogeneity existed in the tools used within each domain (Fig. 1). Across studies assessing memory, 11 distinct instruments were employed, while 10 different tools were used to evaluate executive functions. Within these domains, the most frequently used measures were forward and backward Digit Span for memory (26.7% of studies) and the Trail Making Test (TMT) for executive functions (36.8%). Similarly, considerable variability was evident in domains assessing attention, psychomotor functions, and perceptual speed, with more than five different instruments used in each domain. The most commonly applied tools were the Gradual Continuous Performance Test (GCPT) for attention (33.3%), the Digit Symbol Substitution Test (DSST) for psychomotor functions (37.5%), and Symbol Search for perceptual speed (33.3%). Global cognition was typically assessed using the Mini-Mental State Examination (MMSE) or Montreal Cognitive Assessment (MoCA), each accounting for 38.5% of assessments; studies using MMSE solely as a screening tool were excluded from this count.[19, 33] In domains assessed infrequently (perceptual, language, perceived cognitive function), no tool was used more than once.

Across commonly examined CGM metrics (TBR, TAR, CV, mean glucose, TIR, SD, and MAGE), the distribution of cognitive domains was highly consistent (See supplementary material Fig. 4). Executive functions, memory, attention, thought functions, and intellectual functions consistently accounted for the majority of cognitive assessments—showing nearly identical patterns across metrics. In contrast, psychomotor, language, and perceived cognitive domains remained infrequently evaluated regardless of the CGM metric.

## Discussion

The present scoping review evaluated the feasibility of CGM implementation in cognitive research and mapped the available evidence to identify knowledge gaps. A total of 21 studies met the inclusion criteria, with 16 of these published within the past four years, highlighting the rapidly growing adoption of CGM in cognitive research among diabetes populations. Across studies that reported adherence, CGM data completeness was consistently high, supporting the feasibility of using CGM in cognitive research among individuals with diabetes. However, the included studies were highly heterogeneous with respect to study design, CGM-derived metrics, and cognitive assessment tools, and several important knowledge gaps were identified.

Among the 20 observational studies included, only two employed longitudinal follow-up of 1–2 years. Most studies instead monitored glucose over days to weeks, making them well suited to examine acute glucose–cognition relationships. However, these study designs cannot determine whether glycemic patterns contribute to progressive cognitive decline or cognitive trajectories over time. Given the progressive nature of cognitive decline, [38] longitudinal studies are essential to clarify the temporal relationships between glycemic dynamics and cognitive changes. In addition, only four studies leveraged EMA to capture cognitive performance in daily life. This repeated-measures approach enables evaluation of within-person temporal associations between glucose fluctuations and deviations from an individual’s usual cognitive performance, moving beyond static between-person comparisons and providing stronger evidence for temporally proximal associations. Although still observational, EMA-based designs represent an important step toward clarifying potential causal mechanisms operating on short time scales. Notably, all EMA-based studies were conducted in individuals with T1DM. The absence of EMA studies in T2DM populations represents a major gap, particularly given the high prevalence of T2DM and its substantial contribution to diabetes-related cognitive burden.[39] Future EMA-based research in T2DM could substantially advance precision monitoring and personalized intervention strategies by capturing real-time glucose–cognition interactions.

The review revealed that several studies in T2DM restricted their samples to older adults and/or excluded individuals with cognitive impairment or dementia. This is a critical gap because diabetes-related cognitive changes can emerge as early as midlife, [40] so broader age inclusion is necessary. Additionally, more research is needed using CGM in people with coexisting diabetes and mild cognitive impairment or Alzheimer’s disease and related dementias. CGM use is underutilized in these populations despite being associated with improved survival in real-world settings.[41] Studying such high-risk groups may reveal whether glucose–cognition dynamics differ from those observed in cognitively intact individuals with diabetes, and it may guide tailored glucose management strategies for those with existing cognitive impairment.

Notably, most study samples were predominantly White, with limited racial and ethnic diversity. Yet racial and ethnic minority individuals are more likely to have poor glycemic control than non-Hispanic White individuals, [42] and diabetes technologies such as CGM — which can improve glucose control — are less frequently used in these groups.[43] Without intentional inclusion of underrepresented racial and ethnic groups, existing inequities in diabetes care and cognitive health could be perpetuated. Future research should explicitly seek to include diverse populations and expand equitable access to CGM to help reduce cognitive health disparities in diabetes. As the evidence base grows, it could also inform policy changes (e.g., insurance coverage expansions) to improve CGM access for traditionally underserved groups.

This scoping review highlighted substantial heterogeneity in both the CGM-derived metrics and the cognitive assessment tools used across studies. Across the 21 studies, 19 distinct CGM metrics were reported, spanning range-based indices, variability measures, composite scores, and postprandial indicators. However, most studies relied heavily on a narrow subset of traditional metrics such as TIR, TBR, TAR, CV, SD, and MAGE. More advanced measures—such as the Complexity of Glucose Time Series Index (CGI), which captures the adaptability and regulatory integrity of glucose dynamics across time—were rarely applied, even though one study found that a complexity index had a more pronounced association with cognitive function than traditional metrics in people with T2DM.[33] More consistent use of advanced CGM metrics in future work may provide deeper insight into the relationships between glucose fluctuations and specific cognitive outcomes.

Similarly, variability was observed in the assessment of cognitive function. Even within the same cognitive domains, a wide array of instruments was used. For example, memory and executive function were each assessed using numerous non-overlapping tests across different studies. This lack of standardization complicates the synthesis of findings and impedes the development of clinically actionable conclusions. Furthermore, certain cognitive domains such as perceptual function, psychomotor function, language, and perceived cognitive function were infrequently evaluated, even though individuals with diabetes can experience declines in these areas [18, 44, 45] Given that perceived cognitive function is associated with diabetes self-management and overall quality of life, [18] future CGM-based studies should incorporate both objective cognitive measures and subjective cognitive outcomes.

Taken together, the considerable variability in glucose metrics and cognitive measurement approaches underscores the need for greater methodological harmonization in this field. The use of a core set of standardized CGM metrics, combined with validated and sensitive cognitive assessments—including both established batteries (e.g., the NIH Toolbox) and ambulatory, smartphone-based measures (e.g., Sliwinski’s ambulatory cognitive tests or NeuroUX platforms)—would enhance reproducibility, improve cross-study comparability, and increase sensitivity to subtle, early cognitive changes in diabetes. Notably, despite the wide variability in CGM metrics, there was relative consistency in the cognitive domains examined across studies, indicating that researchers have tended to focus on similar cognitive targets regardless of the specific glucose measures employed. In other words, glucose dysregulation in diabetes is often conceptualized as having a general impact on cognition rather than selectively affecting specific domains. Moving forward, it will be important for studies to explicitly test whether distinct CGM-derived patterns are differentially related to particular cognitive domains, rather than assuming a uniform effect on cognition.

Importantly, beyond its research utility, CGM use has demonstrated clear clinical benefits, including improvements in overall glycemic control and reductions in glycemic variability[46] as well as enhanced diabetes self-management behaviors.[47] These established benefits provide further support for integrating CGM into cognitive research, since improved glucose regulation itself may be a modifiable pathway for preserving cognitive health in individuals with diabetes.

To our knowledge, this scoping review provides the first comprehensive mapping of CGM implementation in cognitive research among individuals with diabetes. Nonetheless, several limitations should be acknowledged. First, we did not systematically search gray literature or unpublished studies, raising the possibility of publication bias. Second, data extraction relied on information reported in the published articles, and variability in reporting quality may have led to misclassification or underrepresentation of certain study characteristics. Finally, because this review was designed to map the scope and methodologies of the existing literature rather than to quantify effect sizes or test causality, we did not perform a quantitative synthesis of findings. These limitations underscore the need for future systematic reviews and meta-analyses that can combine data across studies, quantify associations between CGM metrics and cognitive outcomes, and evaluate causality. By addressing these gaps in the literature, future research can better inform clinical strategies to leverage CGM technology for cognitive health in diabetes.

## Conclusions

This scoping review demonstrates that CGM is not only feasible but also increasingly applied in cognitive research involving individuals with diabetes. To advance the field, future studies should prioritize longitudinal designs and adopt broader, more inclusive sampling strategies that reflect the diversity of the diabetes population across age, cognitive status, and racial and ethnic backgrounds. Clarifying which CGM-derived metrics are most robustly associated with specific cognitive domains will be essential for generating clinically meaningful insights. Greater methodological standardization, including the adoption of validated cognitive assessment tools and consensus-based CGM metrics, is urgently needed to enhance reproducibility, reduce measurement bias, and facilitate evidence synthesis. Additionally, integrating EMA offers a unique opportunity to capture real-time, within-person glucose–cognition dynamics in daily life. Integrating CGM more systematically into cognitive research may not only deepen mechanistic insights but also inform the development of personalized interventions aimed at preserving cognitive health through optimized glucose management in people with diabetes.

## Key References

- Cuevas H, Heitkemper E, Haque B. Relationships Among Perception of Cognitive Function, Diabetes Self-Management, and Glucose Variability in Older Adults A Mixed Methods Study. Research in Gerontological Nursing. 2022;15(4):203 − 12.
  - ◌ This study used CGM to examine associations among perceived cognitive function, glucose variability, and diabetes self-management in older adults with type 2 diabetes. It is particularly important because the sample included a relatively larger proportion of racial and ethnic minority participants than is typical in this literature, helping extend the relevance of this work to more diverse populations.
- Cuevas H, Stuifbergen AK, Hilsabeck R, Kim J, Wood S. Perceived Cognitive Function and Glycemic Variability: Baseline Results From a Cognitive Rehabilitation Intervention. Sci Diabetes Self Manag Care. 2024;50(4):310-9.
  - ◌ This study provides important preliminary evidence linking perceived cognitive function with glycemic variability in adults with type 2 diabetes. It is especially relevant because it helps support the clinical significance of perceived cognitive functioning as an outcome in diabetes research and highlights the potential importance of glycemic dynamics in everyday cognitive experience.
- Soholm U, Broadley M, Zaremba N, Divilly P, Baumann PM, Mahmoudi Z, et al. The impact of hypoglycaemia on daily functioning among adults with diabetes: a prospective observational study using the Hypo-METRICS app. Diabetologia. 2024;67(10):2160-74.
  - ◌ This study is highly important because it prospectively followed adults with diabetes for 70 days using CGM and app-based data collection to examine the impact of hypoglycemia on daily functioning in real-world settings. It demonstrates the value and feasibility of longitudinal, intensive monitoring approaches for capturing the day-to-day consequences of glycemic events outside the clinic.

## Supplementary Information

Below is the link to the electronic supplementary material.

Supplementary Material 1 (DOCX 17.0 KB)

Supplementary Material 2 (DOCX 16.7 KB)

Supplementary Material 3 (DOCX 24.6 KB)

Supplementary Material 4 (DOCX 85.6 KB)

Supplementary Material 5 (DOCX 59.6 KB )

Supplementary Material 6 (DOCX 80.2 KB)

Supplementary Material 7 (DOCX 82.1 KB)

Supplementary Material 8 (DOCX 100 KB)

## Data Availability

No datasets were generated or analyzed during the current study.
