# Supplementary material for: Applications of Continuous Glucose Monitoring in Cognitive Research in Diabetes: a Scoping Review
Source: Curr Diab Rep. 2026 Aug 6;26(1):24. doi: 10.1007/s11892-026-01638-1 (PMC13447455; doi:10.1007/s11892-026-01638-1)
Supplement: Supplementary file 1 [file 11892_2026_1638_MOESM1_ESM.docx]

**Supplementary Table 1.**

**PubMed search strategy**

| 1 | "continuous glucose monitoring"[tiab] OR "CGM"[tiab] OR "glucose sensor"[tiab] OR "glucose monitoring"[tiab] OR "Blood Glucose Self-Monitoring"[MeSH Terms] |
| --- | --- |
| 2 | "cognition"[tiab] OR "cognitive function"[tiab] OR "cognitive performance"[tiab] OR "cognitive impairment"[tiab] OR "cognitive decline"[tiab] OR "neurocognitive"[tiab] OR "neuropsychological"[tiab] OR "executive function"[tiab] OR "memory"[tiab] OR "attention"[tiab] OR "Cognition"[MeSH Terms] OR "Neuropsychological Tests"[MeSH Terms] |
| 3 | "diabetes"[tiab] OR "diabetes mellitus"[tiab] OR "type 1 diabetes"[tiab] OR "type 2 diabetes"[tiab] OR "T1DM"[tiab] OR "T2DM"[tiab] OR "Diabetes Mellitus"[MeSH Terms] |

**CINAHL search strategy**

| 1 | MH "Blood Glucose Self-Monitoring" OR "continuous glucose monitoring") |
| --- | --- |
| 2 | MH "Cognition" OR MH "Cognition Disorders" OR "cognitive function" OR "cognitive decline" OR "executive function" OR memory OR attention OR neuropsychological |
| **3** | MH "Diabetes Mellitus" OR diabetes OR "type 1 diabetes" OR "type 2 diabetes" OR T1DM OR T2DM |

Cochrane Library, EMBASE, and PsycINFO were searched using similar strategies
