# Supplementary material for: Applications of Continuous Glucose Monitoring in Cognitive Research in Diabetes: a Scoping Review
Source: Curr Diab Rep. 2026 Aug 6;26(1):24. doi: 10.1007/s11892-026-01638-1 (PMC13447455; doi:10.1007/s11892-026-01638-1)
Supplement: Supplementary file 2 [file 11892_2026_1638_MOESM2_ESM.docx]

| Category | Full Name | Definition |
| --- | --- | --- |
| Central Tendency Measures | Mean Glucose Level | The average glucose concentration over the CGM monitoring period. |
| Range-Based Glycemic Control | Time in Range (TIR) | Percentage of time spent within the target glucose range (70–180 mg/dL). |
|  | Time Above Range (TAR) | Percentage of time above the upper limit of the target range (>180 mg/dL). |
|  | Time Below Range (TBR) | Percentage of time below the lower limit of the target range (<70 mg/dL). |
|  | Time in Tight Range (TITR) | Percentage of time within a narrower glucose range (e.g., 70–140 mg/dL). |
|  | Percent Outside Range (% Out of TIR) | Combined percentage of time outside target range (TAR + TBR). |
|  | Time Above 12 mmol/L (Time > 12 mmol/L) | Percentage of time with glucose exceeding 12 mmol/L (~216 mg/dL). |
|  | Time in Very High (TVH) | Percentage of time glucose exceeds 250 mg/dL. |
| Glycemic Variability | Standard Deviation (SD) | Absolute variability in glucose concentration. |
|  | Coefficient of Variation (CV) | Relative variability calculated as SD ÷ mean glucose × 100. |
|  | Mean Amplitude of Glycemic Excursions (MAGE) | Average amplitude of major glucose fluctuations exceeding a predefined threshold. |
|  | Largest Amplitude of Glucose Excursion (LAGE) | Largest single rise or fall observed in glucose levels. |
|  | Mean of Daily Differences (MODD) | Mean absolute difference between glucose values at the same time on successive days; reflects day-to-day variability. |
|  | Multi-Scale Glycemic Variability (Multi-Scale GV) | Measures glycemic variability across multiple temporal scales by decomposing CGM data using the Ensemble Empirical Mode Decomposition algorithm; variability is quantified as the standard deviation of each intrinsic mode function. |
| Postprandial Glycemic Response | Postprandial Glycemia (PPG) | Glucose concentration or glucose rise measured after meal ingestion. |
|  | Maximum Postprandial Peak Glucose (Gmax) | Highest glucose value observed during the postprandial period. |
| Composite Glycemic Indicators | 24-hour Area Under the Curve (AUC_24_) | Represents the total area under the CGM glucose curve over a 24-hour period, reflecting overall daily glucose exposure. |
|  | Glycemic Risk Index (GRI) | Composite score integrating hypo- and hyperglycemia risk. |
|  | Complexity of Glucose Time Series Index (CGI) | Quantifies the multiscale complexity of glucose fluctuations; lower values indicate more regular and less complex glucose dynamics. |

**Supplementary Table S2.**
