# Supplementary material for: Applications of Continuous Glucose Monitoring in Cognitive Research in Diabetes: a Scoping Review
Source: Curr Diab Rep. 2026 Aug 6;26(1):24. doi: 10.1007/s11892-026-01638-1 (PMC13447455; doi:10.1007/s11892-026-01638-1)
Supplement: Supplementary file 3 [file 11892_2026_1638_MOESM3_ESM.docx]

**Supplementary Table S3.**

| **Cognitive domains** | **ICF code** |  | **Included studies No. (1–21)** |
| --- | --- | --- | --- |
| **Global Mental Functions** |  |  |  |
| **Intellectual functions** | b117 | Global cognitive function | 1, 6, 9, 11, 14, 16, 17, 20 |
| **Specific Mental Functions** |  |  |  |
| **Attention functions** | b140 | Attention | 4, 6, 9, 16, 18, 19 |
|  |  | Sustained attention | 7, 8, 13, 21 |
| **Memory functions** | b144 | Memory | 4, 6, 16 |
|  |  | Immediate memory | 6, 14, 19 |
|  |  | Delayed memory (recall) | 6, 19 |
|  |  | Cued recall | 6 |
|  |  | Long-delayed recognition | 6 |
|  |  | Verbal recognition memory | 2 |
|  |  | Verbal learning and memory | 5 |
|  |  | Visual memory | 5, 9 |
|  |  | Short-term memory | 12 |
|  |  | Working memory | 12, 16, 18 |
|  |  | Visuospatial working memory | 21 |
| **Psychomotor functions** | b147 | Psychomotor speed and concentration | 12 |
|  |  | Visuomotor speed | 14 |
|  |  | Psychomotor processing speed | 21 |
|  |  | Visual-motor coordination | 2 |
| **Perceptual functions** | b156 | Visuospatial ability | 5, 6 |
|  |  | Visuospatial construction ability | 19 |
|  |  | Perceptual speed | 8, 13 |
| **Thought functions** | b160 | Processing speed | 2, 4, 7, 9, 12, 16, 18, 19 |
| **Higher-level cognitive functions** | b164 | Executive function | 1, 2, 4, 5, 6, 12, 14, 16, 18, 19, 20 |
|  |  | Executive function and decision-making | 9 |
|  |  | Cognitive flexibility | 14 |
|  |  | Response inhabitation | 21 |
| **Mental functions of language** | b167 | Language | 6, 19 |
|  |  | Verbal fluency | 16 |
