# Supplementary material for: Applications of Continuous Glucose Monitoring in Cognitive Research in Diabetes: a Scoping Review
Source: Curr Diab Rep. 2026 Aug 6;26(1):24. doi: 10.1007/s11892-026-01638-1 (PMC13447455; doi:10.1007/s11892-026-01638-1)
Supplement: Supplementary file 5 [file 11892_2026_1638_MOESM5_ESM.docx]

Figure 1. Flow diagram of the selection process.

Studies from databases/registers **(n = 1,589)**

CINAHL (n = 78)

PubMed (n = 682)

Cochrane Library (n = 323)

Embase (n = 450)

PsycInfo (n= 56)

References from citation searching **(n = 4)**

**Identification**

Studies excluded **(n = 1,307)**

Studies screened **(n = 1,342)**

Duplicates identified by EndNote **(n = 251)**

Studies excluded **(n = 14)**

- **Abstract (n = 1)**
- **Not a diabetes population (n= 4)**
- **Replicate study (n = 2)**
- **Pediatric population (n = 3)**
- **No cognitive outcome (n = 4)** populations

pediatric population

Full-text assessed for eligibility **(n = 35)**

**Screening**

**Included**

Studies included in review **(n = 21)**
