# Supplementary material for: Applications of Continuous Glucose Monitoring in Cognitive Research in Diabetes: a Scoping Review
Source: Curr Diab Rep. 2026 Aug 6;26(1):24. doi: 10.1007/s11892-026-01638-1 (PMC13447455; doi:10.1007/s11892-026-01638-1)
Supplement: Supplementary file 8 [file 11892_2026_1638_MOESM8_ESM.docx]

Figure 4. Cross-tabulation of CGM Metrics and Cognitive Domains
